# Integrated Genomic and Functional Characterization of Palmitoylation in Clear Cell Renal Cell Carcinoma

**DOI:** 10.1155/humu/4647115

**Published:** 2025-11-29

**Authors:** Dong Zhang, Ke Zhang, Minghao Deng, Jiakang Ma, Jian Zhu, Shuijie Shen, Jianjun Xie, Chao Chen

**Affiliations:** ^1^Department of Urology, Nantong Hospital of Traditional Chinese Medicine, Affiliated Traditional Chinese Medicine Hospital of Nantong University, Nantong, China; ^2^Clinical Medical Research Center, Nantong Hospital of Traditional Chinese Medicine, Affiliated Traditional Chinese Medicine Hospital of Nantong University, Nantong, China; ^3^Department of Urology, The Affiliated Suzhou Hospital of Nanjing Medical University, Suzhou Municipal Hospital, Suzhou, China; ^4^Henan Key Laboratory of Cancer Epigenetics, Cancer Institute, The First Affiliated Hospital and College of Clinical Medicine of Henan University of Science and Technology, Luoyang, China; ^5^Department of Oncology, Nantong Hospital of Traditional Chinese Medicine, Affiliated Traditional Chinese Medicine Hospital of Nantong University, Nantong, China; ^6^Department of Urology, Ningbo Municipal Hospital of Traditional Chinese Medicine (TCM), Affiliated Hospital of Zhejiang Chinese Medical University, Ningbo, China

**Keywords:** clear cell renal cell carcinoma, immunotherapy, machine learning, palmitoylation, single-cell RNA sequencing, ZDHHC18

## Abstract

**Background:**

Clear cell renal cell carcinoma (ccRCC) is a highly aggressive cancer with a poor prognosis. Palmitoylation, a posttranslational modification, plays a key role in regulating cancer progression and immune responses. However, its influence on ccRCC prognosis and immune therapy efficacy remains underexplored.

**Methods:**

Multiple publicly available ccRCC datasets were integrated and harmonized through batch effect correction. A prognostic model based on palmitoylation-related genes was constructed using a combination of 101 machine learning algorithms. Single-cell RNA sequencing was employed to analyze cellular heterogeneity within the tumor microenvironment. Genomic profiling, including tumor mutational burden (TMB), copy number variation (CNV), and tumor stemness, was conducted to identify genomic differences between the high- and low-risk groups. Immune infiltration levels were assessed using various algorithms to compare immune profiles across patient subgroups, while immune therapy responses were predicted using multiple prediction models. Experimental validation of ZDHHC18, a key gene in the prognostic model, was performed in ccRCC cell lines (786-O and Caki-1) to evaluate its impact on cell proliferation, migration, and invasion.

**Results:**

The palmitoylation-related prognostic model effectively stratified ccRCC patients into the high- and low-risk groups, with distinct differences in survival outcomes. Genomic analysis demonstrated higher TMB and CNV alterations in the high-risk group. Immune response predictions indicated that low-risk patients were more likely to benefit from immunotherapy. Additionally, ZDHHC18 was significantly upregulated in ccRCC tumor tissues, and its knockdown notably inhibited cell proliferation, migration, and invasion.

**Conclusion:**

Palmitoylation-related genes, particularly ZDHHC18, serve as promising prognostic biomarkers and predictive indicators for immune therapy in ccRCC. These findings offer new insights into ccRCC biology and highlight potential therapeutic targets for improving patient outcomes.

## 1. Introduction

Renal cell carcinoma (RCC), with clear cell renal cell carcinoma (ccRCC) as its predominant subtype, represents the most common and aggressive form of kidney cancer [1]. ccRCC is characterized by its high metastatic potential and resistance to conventional treatments, resulting in a generally poor prognosis for patients [2]. Despite the advent of targeted therapies and immune checkpoint inhibitors, the clinical outcomes of ccRCC patients remain heterogeneous due to the complex molecular and genetic landscape of the disease [3, 4]. As a result, there is an ongoing need for more effective prognostic biomarkers and personalized therapeutic strategies that can improve patient management and clinical outcomes.

Palmitoylation is a dynamic and reversible posttranslational modification in which a palmitic acid molecule (a saturated fatty acid) is covalently attached to cysteine residues of target proteins [5]. This modification is catalyzed by palmitoyltransferases, predominantly the ZDHHC (zinc finger DHHC–type containing) family, and influences a variety of cellular processes, including protein localization, stability, and interaction with cellular membranes [6, 7]. Due to its reversible nature, palmitoylation is a key posttranslational modification that plays a crucial role in regulating various biological functions of proteins, impacting both cellular physiology and pathology. Advances in research technologies have enhanced our understanding of the molecular mechanisms underlying palmitoylation, shedding light on its diverse roles in health and disease. This deeper insight opens up new possibilities for the development of targeted therapeutic strategies [8].

In cancer biology, aberrant palmitoylation has been increasingly recognized as a critical factor in tumorigenesis [9]. Dysregulated palmitoylation impacts key oncogenic pathways by modulating the activity, localization, and interactions of various proteins involved in cell proliferation, migration, survival, and metastasis [10, 11]. In ccRCC, palmitoylation plays a critical role in regulating metabolic reprogramming. Studies have shown that the palmitoyltransferase DHHC9 and the acyl protein thioesterase APT1 modulate the palmitoylation of *β*-catenin, thereby influencing renal fibrosis progression [12]. This mechanism is likely to also impact the metabolic reprogramming of ccRCC, contributing to tumorigenesis and disease progression. In conclusion, the study of palmitoylation in renal cancer highlights its potential as a promising therapeutic target. By modulating metabolic pathways and protein functions, palmitoylation may offer novel insights and strategies for the treatment of renal cancer.

In this study, we explore the role of palmitoylation in ccRCC through an innovative approach by integrating machine learning techniques to identify palmitoylation-related prognostic biomarkers. By combining multiomics data and single-cell RNA sequencing, we aim to enhance the understanding of palmitoylation's impact on ccRCC biology and its potential as a therapeutic target. This work is among the first to use machine learning in the context of palmitoylation to predict prognosis and guide treatment strategies for ccRCC, providing new avenues for precision medicine in renal cancer.

## 2. Methods

### 2.1. Data Integration and Preprocessing

This study integrated multiple publicly available ccRCC transcriptomic datasets, including TCGA (https://portal.gdc.cancer.gov/, *n* = 513), ICGC (https://dcc.icgc.org/, *n* = 91), E-MTAB-1980 (https://www.ebi.ac.uk/arrayexpress/, *n* = 101), GSE73731 (*n* = 265), and GSE40435 (*n* = 101) (https://www.ncbi.nlm.nih.gov/geo/), excluding patients with a survival time of less than 30 days to enhance data integrity. To ensure compatibility across datasets, high-throughput sequencing data were converted to Transcripts Per Million (TPM) format, and genes with an average expression level below 0.5 were excluded to remove lowly expressed genes. The datasets were merged using the “inSilicoMerging” algorithm [13], followed by batch effect removal using the “Combat” algorithm to correct for systematic biases [14]. Additionally, single-cell RNA sequencing data from the TISCH2 database were utilized to explore cellular heterogeneity and gene expression profiles within the tumor microenvironment [15]. Finally, 25 key palmitoylation-related genes were identified by cross-referencing the CellPalmSeq, SwissPalm, and BrainPalmSeq databases, along with relevant literature (Table S1) [9, 11]. The clinical characteristics of patients from the TCGA, ICGC, E-MTAB-1980, GSE73731, and GSE40435 cohorts are summarized in Table S2. Detailed experimental procedures are provided in Table S3.

### 2.2. Integrating Palmitoylation-Related Genes and Machine Learning Analysis

To develop a prognostic model for ccRCC, we used the TCGA dataset as the training cohort and the ICGC and E-MTAB-1980 datasets as validation cohorts. After merging and batch effect correction, we retained the intersection of protein-coding genes across all datasets, normalized gene expression using *Z*-score transformation, and excluded genes with an average TPM below 0.5. Initially, 10 machine learning algorithms were applied in 101 different combinations to identify the optimal model for prognostic prediction. These included random survival forest (RSF), Lasso–Cox, Elastic Net, CoxBoost, GBM, PLS–Cox, SuperPC, survival SVM, and stepwise Cox regression, as well as their paired or sequential integrations (e.g., Lasso + RSF, RSF + Enet, and StepCox + GBM). Each model was trained using the TCGA cohort and validated on the two external datasets. Hyperparameters were tuned via 10-fold cross-validation: For instance, alpha values in Elastic Net were searched from 0 to 1, while RSF and GBM models were optimized based on out-of-bag or cross-validated error. Variable selection was performed using importance measures (e.g., RSF variable importance > 0.4 or nonzero Lasso coefficients). For each model, a patient-specific risk score was calculated from the predicted survival risk. Model performance was evaluated using concordance index (*C*-index), the Kaplan–Meier survival analysis, and time-dependent receiver operating characteristic (ROC) curves. The Lasso regression and RSF combination yielded the highest average *C*-index across cohorts and was selected as the final prognostic model.

### 2.3. Clinical Correlation and Prognostic Independence Analysis

To determine whether the palmitoylation-based risk score is an independent prognostic factor, we performed both univariate and multivariate Cox proportional hazards regression analyses. The analyses incorporated established clinical and pathological variables, including age, tumor grade, T stage, N stage, M stage, and overall stage, alongside the risk score derived from the gene signature. These analyses were conducted using the “survival” R package. To further assess the predictive accuracy of the model, calibration curves were plotted using the “rms” R package to compare the predicted survival probabilities with the actual observed outcomes. In addition, we conducted decision curve analysis (DCA) using the “rmda” package to evaluate the clinical net benefit of the risk score compared with conventional prognostic indicators across a range of threshold probabilities.

### 2.4. Biological Pathways and Processes Differentiating Two Risk ccRCC Groups

To investigate the underlying biological mechanisms contributing to the prognostic differences between the high- and low-risk groups, we performed comprehensive functional enrichment analyses. Gene Set Variation Analysis (GSVA) was conducted using the hallmark gene sets to identify distinct pathway enrichments between the two groups. Additionally, Gene Ontology (GO) functional enrichment analysis was performed to assess biological process variations, while Kyoto Encyclopedia of Genes and Genomes (KEGG) pathway analysis was carried out to explore key signaling pathways differentially enriched in the high- and low-risk groups. These analyses were categorized according to their biological relevance to further elucidate the potential mechanisms driving the prognosis of ccRCC patients.

### 2.5. Single-Cell Analysis and Pathway Exploration of ZDHHC18

To explore the role of ZDHHC18 in ccRCC at the single-cell level, five publicly available single-cell RNA sequencing datasets (GSE111360, GSE171306, GSE139555, GSE121636, and GSE159115) were obtained from the TISCH2 database. Expression profiles of ZDHHC18 were assessed across different datasets to examine its distribution among distinct cell populations within the tumor microenvironment. For more detailed analysis, two datasets with comprehensive cell-type annotations, GSE171306 and GSE159115, were selected for reclustering and further investigation. Cell types were reannotated based on canonical marker genes. The Seurat R package was used to calculate palmitoylation-related gene set scores using the AddModuleScore function. Expression levels of ZDHHC18 and the corresponding module scores were assessed across annotated cell types. To investigate intercellular communication patterns, the CellChat R package (Version 1.6.1) was applied to infer and quantify cell-to-cell interaction networks within the tumor microenvironment, with a specific focus on signaling interactions involving malignant epithelial cells.

### 2.6. Genomic Landscape and Heterogeneity

To further explore the genomic heterogeneity between the high-risk and low-risk groups, we conducted a series of comprehensive analyses, including tumor mutational burden (TMB), mutation frequency, copy number variation (CNV), and tumor stemness analysis. TMB was quantified using ccRCC samples from the TCGA cohort. The frequencies of all mutations, nonsynonymous mutations, and synonymous mutations were compared between the two groups. CNV analysis was performed to evaluate amplification and deletion frequencies, as well as focal loss and gain loads and broad loss and gain loads. Tumor stemness was assessed using the EREG-mRNAsi index, and its association with the risk score was further analyzed. Finally, we examined genomic heterogeneity indices, such as homologous recombination deficiency (HRD), mutational abundance (MATH), loss of heterozygosity (LOH), and tumor purity, to identify differences between the high-risk and low-risk groups.

### 2.7. Immune Therapy Response Prediction Based on Palmitoylation-Related Model

Immunotherapy-related pathways were identified through a comprehensive literature review, with additional immune-related gene sets sourced from the Gene Set Enrichment Analysis (GSEA) database. Correlation analyses were performed to evaluate the relationship between risk scores and immune pathways. Immune infiltration levels were assessed using the ESTIMATE algorithm, which provides the immune score and ESTIMATE score based on gene expression data [16]. Furthermore, immune cell composition was quantified using both the ssGSEA and Cibersort [17] algorithms to explore differences in immune gene expression between the high- and low-risk groups. To predict the likelihood of immune therapy response, we employed the TIDE (Tumor Immune Dysfunction and Exclusion) algorithm, which assesses immune exclusion and dysfunction [18]. According to TIDE classification criteria, patients with a TIDE score < 0 were defined as responders, while those with a TIDE score > 0 were considered nonresponders. The relationship between risk scores and TIDE-related metrics was also investigated. Finally, the Submap algorithm was used for independent validation of the immune response prediction [19], and its results were compared with the TIDE findings.

### 2.8. Statistical Analysis

Categorical variables were summarized as counts and percentages, and continuous variables were expressed as mean ± standard deviation or used directly for modeling and visualization, depending on their purpose. Group comparisons were performed using the chi-square or Fisher's exact test for categorical variables and the Wilcoxon rank-sum test or ANOVA for continuous variables. A two-sided *p* value < 0.05 was considered statistically significant. All analyses were conducted using R software (Version 4.4.2).

## 3. Results

### 3.1. Batch Effect Correction and Palmitoylation Level Analysis in ccRCC

**Figure 1:**
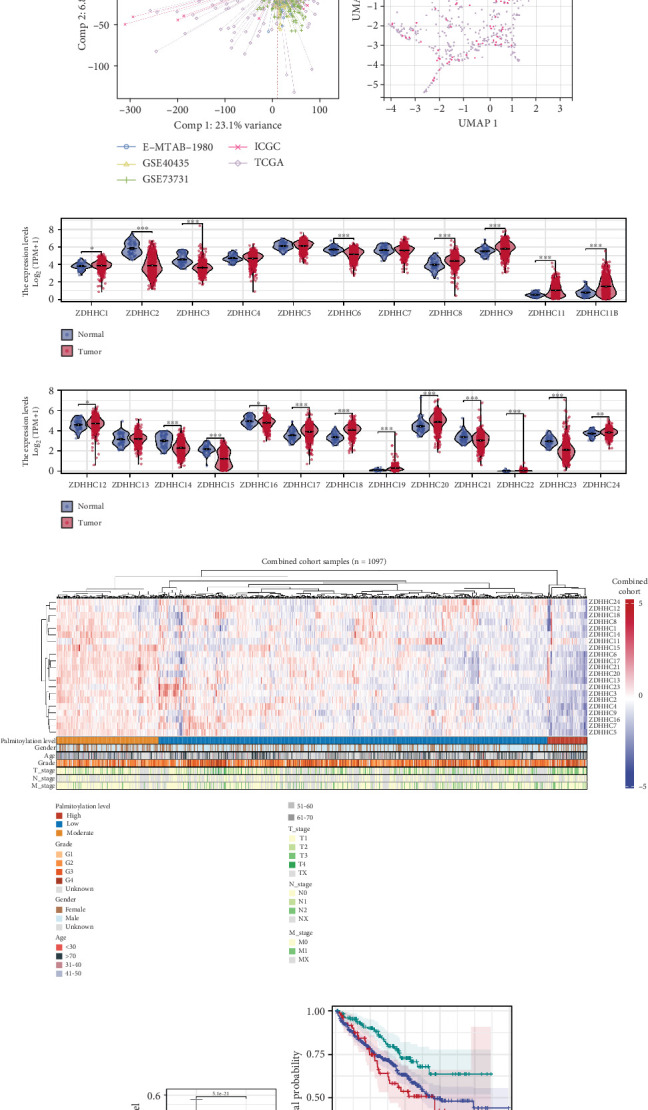
Impact of batch effect correction and palmitoylation alterations in ccRCC. (a) PCA and UMAP plots showing the sample distribution across datasets before batch effect correction, revealing significant differences and indicating the presence of batch effects. (b) PCA and UMAP plots after batch effect correction using the Combat algorithm, showing improved sample clustering across datasets, indicating successful harmonization. (c, d) Differential expression analysis of palmitoylation-related genes between ccRCC and normal samples. (e) Heatmap illustrating the relationship between palmitoylation levels and clinical features in ccRCC samples. (f) Comparison of palmitoylation levels between ccRCC and normal tissues, with significant differences observed. (g) Survival analysis demonstrating distinct prognostic outcomes for patients with different palmitoylation levels. (h) Clinical correlation analysis showing elevated palmitoylation levels in advanced-stage (G4 and T4) and metastatic ccRCC samples.

Prior to batch effect correction, PCA and UMAP plots revealed significant disparities in the sample distributions across datasets, indicating the presence of batch effects (Figure 1a). After batch effect correction with the Combat algorithm, the samples from different datasets clustered together, demonstrating successful harmonization (Figure 1b). Differential expression analysis of palmitoylation-related genes between ccRCC and normal samples showed that most of these genes exhibited significantly altered expression (Figure 1c,d). The palmitoylation levels in ccRCC samples from the TCGA, ICGC, E-MTAB-1980, GSE73731, and GSE40435 cohorts (*n* = 1071) were quantified using the ssGSEA algorithm, and a heat map was generated to visualize the relationship between palmitoylation levels and clinical features (Figure 1e). Notably, ccRCC samples exhibited significantly lower palmitoylation levels compared to normal tissues (Figure 1f). Survival analysis indicated that patients with different palmitoylation levels had significantly distinct prognostic outcomes (Figure 1g). Clinical correlation analysis revealed no significant differences in palmitoylation levels based on age or gender. However, advanced-stage patients (G4 and T4) and those with metastatic ccRCC demonstrated elevated palmitoylation levels (Figure 1h). These findings suggest that palmitoylation levels may influence the progression of ccRCC and that palmitoylation-related genes could represent promising prognostic biomarkers and potential therapeutic targets for ccRCC.

### 3.2. Machine Learning–Determined Prognostic Significance of Palmitoylation-Related Genes in ccRCC

**Figure 2:**
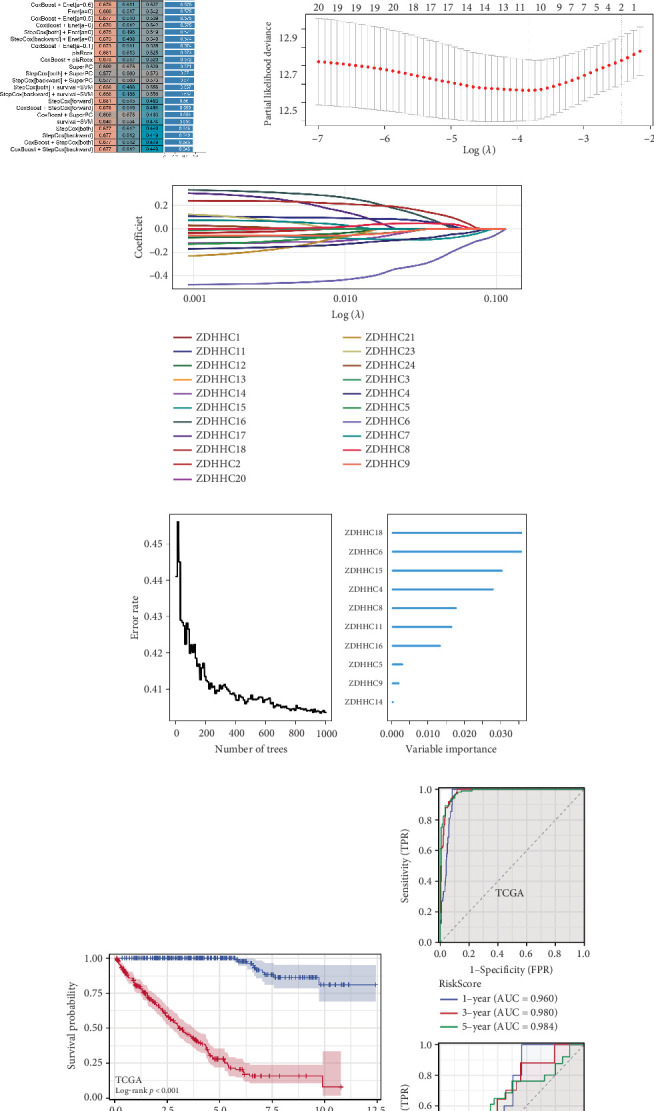
Machine learning–based prognostic model incorporating palmitoylation-related genes. (a) *C*-index values for various combinations of 10 machine learning algorithms, illustrating that the Lasso + RSF combination yielded the highest *C*-index, indicating the most accurate prognostic model. (b, c) Lasso–Cox regression analysis performed using the glmnet algorithm. (d) RSF analysis of the 10 selected genes to calculate the risk score, highlighting ZDHHC18 as the most significant predictor of ccRCC prognosis. (e) Kaplan–Meier survival curves for patients stratified into the high- and low-risk groups based on the median risk score, demonstrating significantly different survival outcomes in both the training and validation sets. (f) Time-dependent ROC curve analysis of the risk score, showing excellent predictive performance with an AUC of 0.99 in the training set.

The prognostic model for ccRCC was constructed using the TCGA dataset as the training set and validated in the ICGC and E-MTAB-1980 datasets. Among the 101 combinations of 10 machine learning algorithms evaluated, the combination of Lasso regression and RSF showed the highest *C*-index, indicating its optimal predictive performance (Figure 2a). Lasso–Cox regression analysis, incorporating survival time, status, and gene expression data, identified 10 key genes after performing 10-fold cross-validation (Figure 2b,c). Further RSF analysis on these 10 genes was conducted to compute a risk score, with ZDHHC18 emerging as the most significant gene in predicting patient outcomes (Figure 2d). Based on the median risk score, patients were stratified into the high-risk and low-risk groups, and the Kaplan–Meier survival analysis demonstrated significantly different survival outcomes between the two groups in both the training set and validation cohorts (Figure 2e). Time-dependent ROC curve analysis showed strong predictive accuracy of the model, with an area under the curve (AUC) of 0.99 in the training set (Figure 2f). To evaluate whether the palmitoylation-based risk score provides independent prognostic information, we conducted both univariate and multivariate Cox proportional hazards regression analyses, including clinical and pathological variables such as age, stage, grade, and TNM classification. The risk score remained a significant independent predictor of overall survival (*p* < 0.001) after adjusting for these covariates (Figure S1A,B). Calibration curves demonstrated good agreement between predicted and observed outcomes (Figure S1C), and DCA confirmed the superior net benefit of the risk score compared to individual clinical features (Figure S1D). These results highlight the potential of palmitoylation-related genes as reliable biomarkers for ccRCC prognosis.

### 3.3. Exploring Functional Pathways and Biological Processes Between Two Risk ccRCC Groups

**Figure 3:**
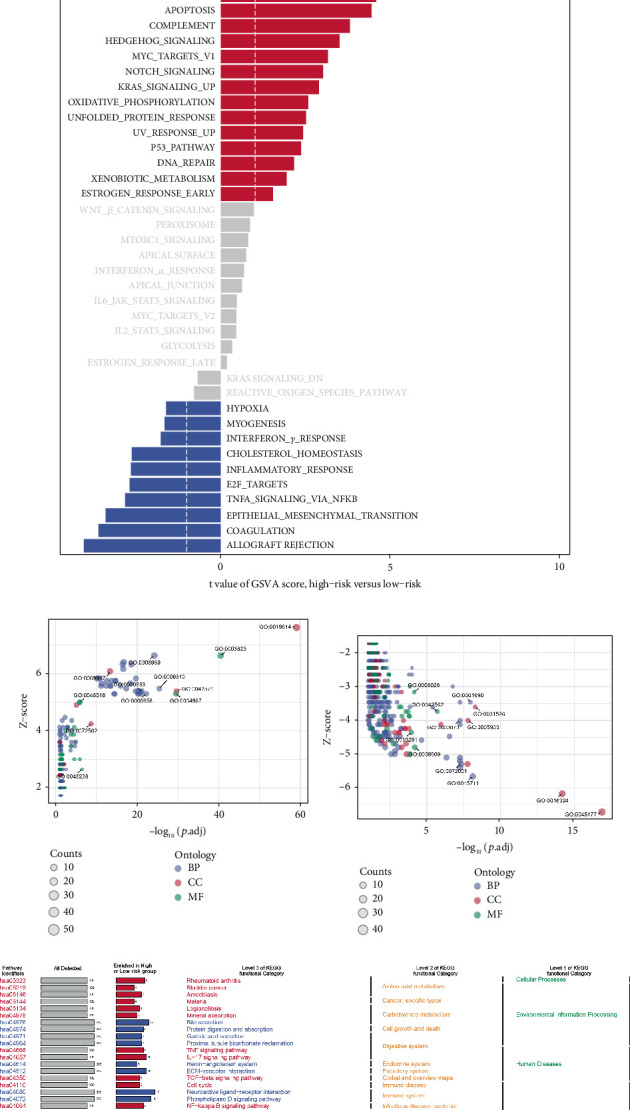
Functional enrichment analysis of the high- and low-risk groups based on the palmitoylation-related model. (a) GSVA analysis of hallmark gene sets, showing distinct pathway enrichments between the high- and low-risk groups. High-risk patients are enriched in pathways related to heme metabolism, PI3K/AKT/mTOR signaling, adipogenesis, and fatty acid metabolism, while low-risk patients show enrichment in pathways such as allograft rejection and coagulation. (b, c) GO functional enrichment analysis highlighting significant differences in biological processes between the high- and low-risk groups. (d) KEGG pathway analysis comparing enriched pathways between the high- and low-risk groups, demonstrating key differences in amino acid metabolism, infectious diseases, cell growth and death, and signal transduction.

GSVA analysis based on the hallmark gene sets revealed significant pathway enrichment differences between the high- and low-risk groups. High-risk patients showed notable enrichment in pathways related to heme metabolism, PI3K/AKT/mTOR signaling, adipogenesis, and fatty acid metabolism, suggesting that these pathways may be associated with poor prognosis and aggressive disease progression (Figure 3a). Conversely, low-risk patients were enriched in pathways such as allograft rejection and coagulation, indicating a potential association with immune response and coagulation processes. GO functional enrichment analysis further supported these findings, showing significant differences in biological processes between the groups (Figure 3b,c). KEGG pathway analysis revealed additional key differences, with high-risk patients displaying enrichment in pathways related to amino acid metabolism and infectious diseases, while low-risk patients were enriched in pathways associated with cell growth, death, and signal transduction (Figure 3d). These results suggest distinct biological processes underlying the prognosis of ccRCC in the high- and low-risk groups.

### 3.4. Single-Cell RNA Profiling Reveals ZDHHC18 Expression and Its Potential Impact on Metabolic Pathways

**Figure 4:**
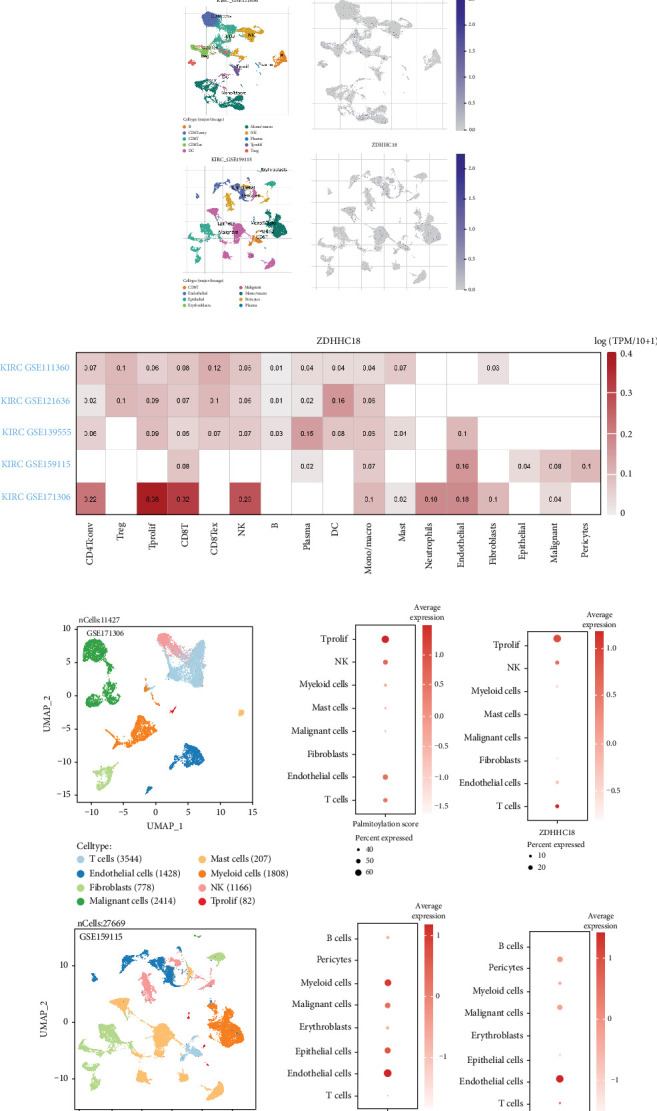
Single cell expression and cell–cell communication analysis of ZDHHC18. (a) Expression distribution of ZDHHC18 across five single-cell datasets (GSE111360, GSE171306, GSE139555, GSE121636, and GSE159115). (b) Expression levels of ZDHHC18 across different immune cell types in GSE171306, with the highest expression observed in Tprolif cells. (c) Bubble plots showing ZDHHC18 expression and palmitoylation-related gene set scores across malignant epithelial subclusters in the GSE171306 and GSE159115 datasets. (d) Number of inferred interactions among different cell populations within the tumor microenvironment.

We assessed the single-cell expression landscape of ZDHHC18 across five ccRCC datasets (GSE111360, GSE171306, GSE139555, GSE121636, and GSE159115) obtained from the TISCH2 database. ZDHHC18 was broadly expressed across multiple cell types within the tumor microenvironment, including immune and stromal populations, with moderate variability among datasets (Figure 4a). While relatively high expression was observed in certain immune subsets, such as Tprolif cells (Figure 4b), our analysis specifically focused on malignant epithelial cells due to the potential oncogenic role of ZDHHC18. To explore this further, we reannotated malignant cell subclusters in the GSE171306 and GSE159115 datasets and evaluated both ZDHHC18 expression and palmitoylation-related gene set scores across these refined subsets. Although ZDHHC18 expression and palmitoylation activity were not selectively elevated in malignant cells, both were consistently detectable within these populations, supporting their potential biological relevance (Figure 4c). In addition, cell–cell communication analysis using the “CellChat” package revealed complex interaction networks involving malignant epithelial cells and other cell types in the tumor microenvironment, providing further context for understanding the functional implications of ZDHHC18 in ccRCC (Figure 4d).

### 3.5. Comprehensive Mutation and Genomic Analysis Between Two Risk ccRCC Groups

**Figure 5:**
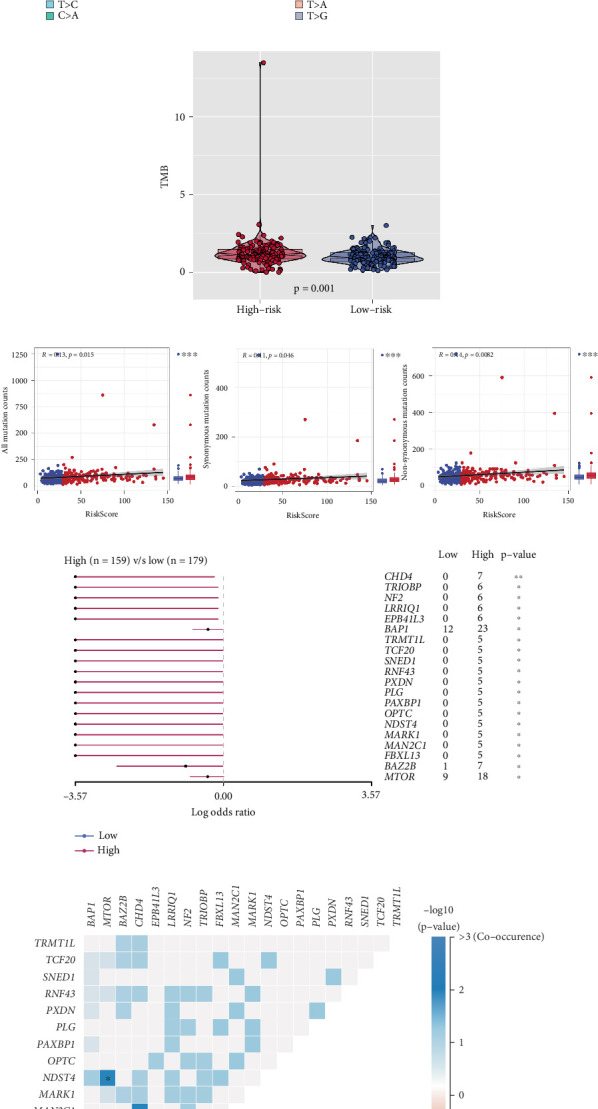
Characterization of mutational burden and genetic alterations. (a) Tumor mutational burden (TMB) in pan-cancer samples, with ccRCC exhibiting a relatively low TMB. (b) Comparison of TMB between the high-risk and low-risk groups, showing significantly elevated TMB in the high-risk group. (c) Comparison of mutation counts between the high- and low-risk groups, highlighting significantly higher mutation rates in the high-risk group for all mutation types, including nonsynonymous and synonymous mutations. (d) Differentially mutated genes between the high- and low-risk groups, with genes like CHD4, TRIOBP, NF2, LRRIQ1, and EPB41L3 found exclusively in the high-risk group. (e) Co-occurrence and mutual exclusion heat map of the top 20 differential mutation genes.

**Figure 6:**
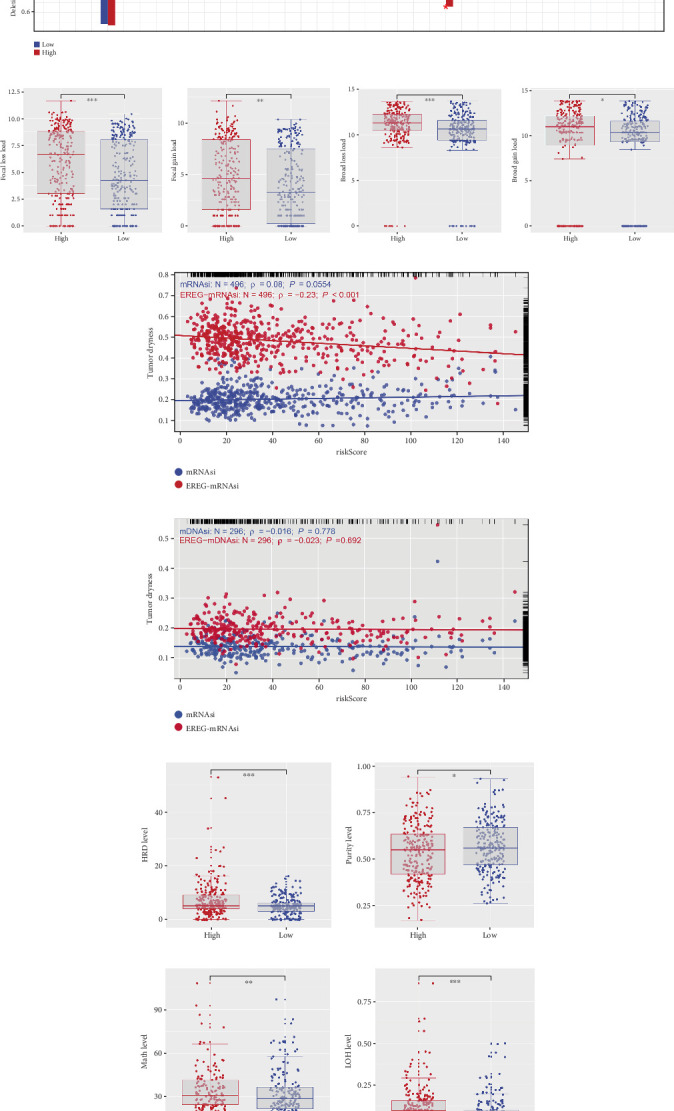
Differential genomic profiles between the two risk groups. (a) Comparison of amplification and deletion frequencies between the high- and low-risk groups. (b) Differences in focal loss load, focal gain load, broad loss load, and broad gain load between the high- and low-risk groups, with significantly higher CNV alterations observed in the high-risk group. (c, d) Negative correlation between risk score and tumor stemness index (EREG-mRNAsi). (e–h) Genomic heterogeneity indices, including HRD, MATH, LOH, and tumor purity, showing significant differences between the high- and low-risk groups, with the high-risk group exhibiting higher levels of genomic instability.

TMB analysis indicated that ccRCC exhibited relatively low mutational burden when compared to other cancer types. However, patients in the high-risk group demonstrated significantly higher TMB levels than those in the low-risk group (Figure 5a,b). Furthermore, the high-risk group had notably higher counts of all mutations, nonsynonymous mutations, and synonymous mutations compared to the low-risk group (Figure 5c). Differential mutation analysis revealed that genes such as CHD4, TRIOBP, NF2, LRRIQ1, and EPB41L3 were exclusively mutated in the high-risk group (Figure 5d). A co-occurrence and mutual exclusion heat map for the top 20 differentially mutated genes demonstrated significant patterns of genetic interaction (Figure 5e). In terms of CNV, the high-risk group exhibited significantly higher focal loss load, focal gain load, broad loss load, and broad gain load when compared to the low-risk group (Figure 6a,b). Additionally, a negative correlation was observed between the risk score and the tumor stemness index (EREG-mRNAsi), indicating a potential link between increased stemness and higher risk (Figure 6c,d). Finally, genomic heterogeneity indices, including HRD, MATH, LOH, and tumor purity, exhibited significant differences between the two groups, with the high-risk group showing a higher degree of genomic instability (Figures 6e, 6f, 6g, and 6h). These results highlight the significant genomic differences between the high- and low-risk ccRCC groups, suggesting that genomic instability and mutational burden play a critical role in the progression and poor prognosis of ccRCC.

### 3.6. Prediction of Immune Therapy Response Based on Palmitoylation-Related Model

**Figure 7:**
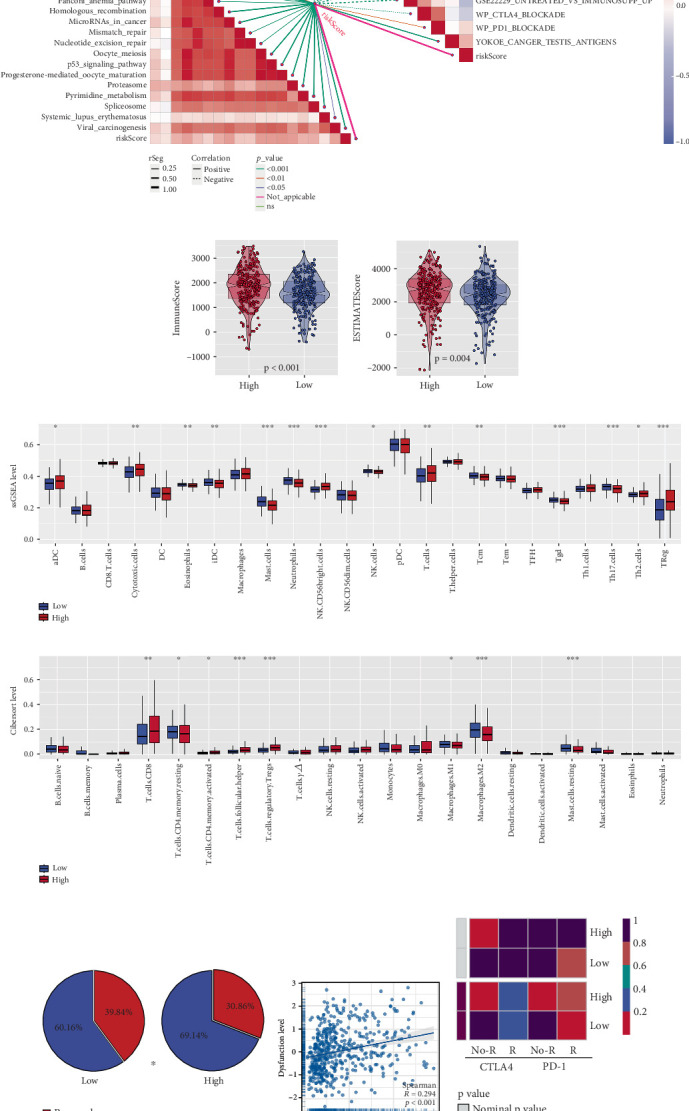
Immune therapy response prediction based on palmitoylation-associated prognostic model. (a) Correlation analysis between the palmitoylation-based risk score and immune therapy–related pathways, showing significant positive correlations with most pathways. (b, c) Immune infiltration analysis using the ESTIMATE algorithm, comparing the immune score and ESTIMATE score between the high-risk and low-risk groups. (d, e) Differential expression of immune-related genes based on ssGSEA and Cibersort algorithms, highlighting significant differences in immune profiles between the two risk groups. (f) TIDE analysis comparing immune therapy response predictions between the high-risk and low-risk groups, indicating a higher proportion of responders in the low-risk group. (g) Correlation between the palmitoylation-based risk score and immune dysfunction levels. (h) Validation of immune therapy response predictions using the Submap algorithm, with results consistent with TIDE analysis, showing better outcomes for low-risk patients.

Correlation analysis revealed that the risk score was positively correlated with the majority of immune therapy–related pathways (Figure 7a). The ESTIMATE algorithm showed that both the immune score and the ESTIMATE score were significantly higher in the high-risk group compared to the low-risk group (Figure 7b). Additionally, differential expression analysis of immune-related genes, quantified by ssGSEA and Cibersort, demonstrated significant differences between the two groups in terms of immune signatures (Figure 7d,e). TIDE analysis further confirmed that a higher proportion of patients in the low-risk group were predicted to respond favorably to immune therapy (Figure 7f). Notably, a positive correlation between risk score and immune dysfunction levels was observed, suggesting that high-risk patients may have an impaired immune response (Figure 7g). Consistent with the TIDE findings, the Submap analysis also indicated that low-risk patients are more likely to benefit from immunotherapy (Figure 7h). These findings suggest that the palmitoylation-related risk score may serve as a useful biomarker for predicting immune therapy response in ccRCC patients, with low-risk patients exhibiting a higher likelihood of benefiting from immune checkpoint inhibition.

### 3.7. ZDHHC18 Expression and Its Biological Role in ccRCC Cells

**Figure 8:**
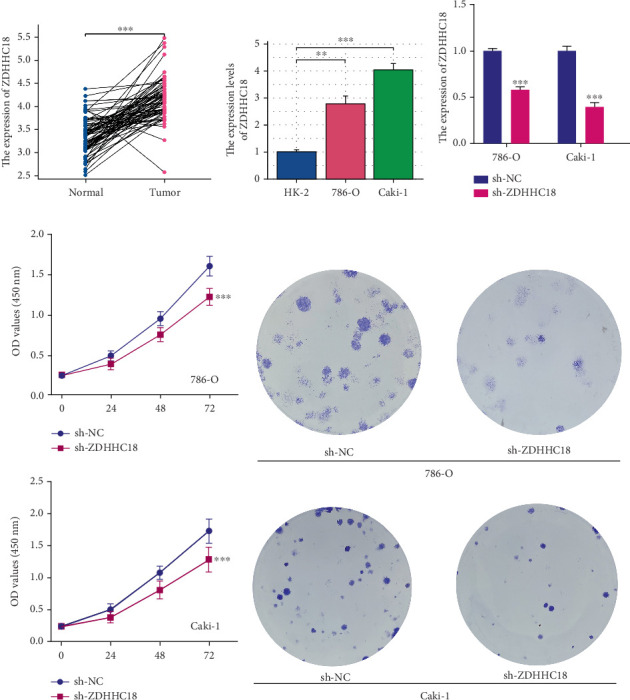
ZDHHC18 expression and functional analysis in ccRCC cells. (a) Expression levels of ZDHHC18 in ccRCC tumor and paired normal tissues from TCGA, showing significant overexpression in tumor tissues. (b) qPCR analysis of ZDHHC18 expression in ccRCC cell lines (786-O and Caki-1) compared to normal renal cells, with significant upregulation in both ccRCC cell lines. (c) qPCR validation of ZDHHC18 knockdown efficiency in stable knockdown ccRCC cell lines. (d, e) CCK-8 and colony formation assays indicating that ZDHHC18 knockdown significantly inhibits ccRCC cell proliferation.

**Figure 9:**
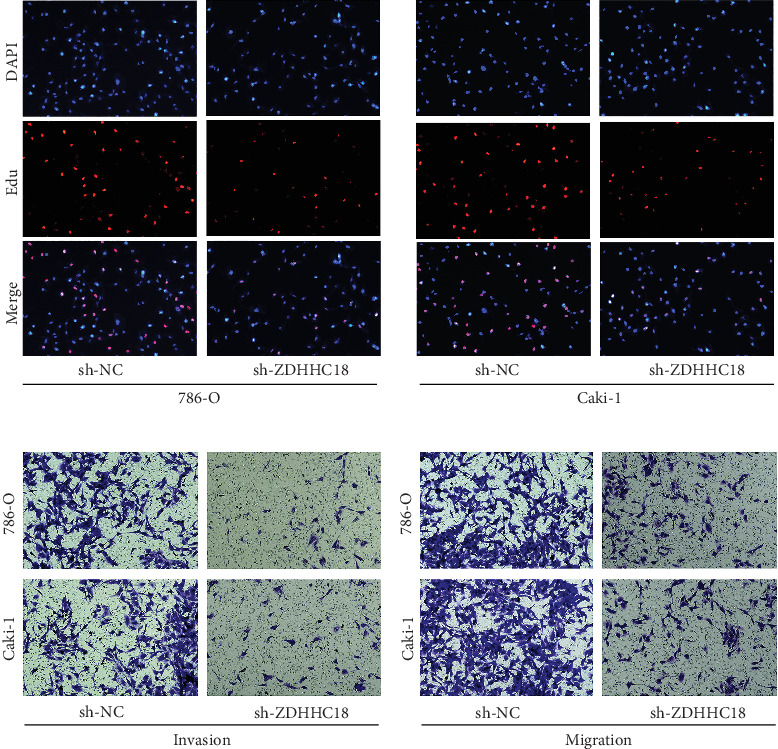
ZDHHC18 knockdown inhibits proliferation, migration, and invasion of ccRCC cells. (a) EdU incorporation assay demonstrating a marked reduction in DNA synthesis in ZDHHC18 knockdown ccRCC cells. (b) Transwell migration and invasion assays showing a significant reduction in the migration and invasion capabilities of ZDHHC18 knockdown ccRCC cells.

Analysis of paired samples from TCGA revealed that ZDHHC18 was significantly overexpressed in ccRCC tumor tissues compared to adjacent normal tissues (Figure 8a). To further validate these findings, we examined ZDHHC18 expression in ccRCC cell lines, including 786-O and Caki-1, and observed a marked upregulation of ZDHHC18 in both cell lines compared to normal renal cells (Figure 8b). We then constructed stable ZDHHC18 knockdown cell lines, with knockdown efficiency confirmed by qPCR (Figure 8c). Functional assays demonstrated that ZDHHC18 knockdown significantly inhibited ccRCC cell proliferation, as evidenced by CCK-8 and colony formation assays (Figure 8d,e). Consistent results were obtained from EdU assays, which showed a significant reduction in DNA synthesis in ZDHHC18 knockdown cells (Figure 9a). Furthermore, Transwell migration and invasion assays revealed that ZDHHC18 knockdown notably suppressed the migratory and invasive abilities of ccRCC cells (Figure 9b). To evaluate whether the functional role of ZDHHC18 is specific to malignant cells, we conducted knockdown experiments in the nontumorigenic HK-2 cell line. qRT-PCR confirmed efficient silencing of ZDHHC18 (Figure S2A). CCK-8 and wound healing assays showed no significant changes in cell proliferation or migration following knockdown (Figure S2B,C), indicating a minimal role for ZDHHC18 in normal epithelial cell function.

## 4. Discussion

In this study, we explored the role of palmitoylation in ccRCC, identifying its potential as both a prognostic marker and a therapeutic target. Through a combination of machine learning, multiomics analysis, and experimental validation, we developed a palmitoylation-related prognostic model that successfully stratified ccRCC patients into the high- and low-risk groups, with significant differences in survival outcomes. Our findings suggest that palmitoylation plays a pivotal role in regulating metabolic reprogramming and tumor progression in ccRCC, providing a basis for novel therapeutic strategies targeting this modification.

Emerging evidence supports a pivotal role of protein palmitoylation in tumor progression, immune evasion, and therapy resistance. A recent review highlights that aberrant S-palmitoylation regulates T-cell activation, cytokine signaling, autophagy, and immune checkpoint pathways, thereby shaping the tumor immune landscape and mediating resistance to therapy [20]. More specifically, in ccRCC, ZDHHC2 has been shown to catalyze palmitoylation of acylglycerol kinase (AGK), promoting its membrane localization and activation of the PI3K/AKT/mTOR axis, which contributes to sunitinib resistance in both cell line and murine models [21]. Though our focus is ZDHHC18, these findings suggest a plausible mechanism by which ZDHHC18-mediated palmitoylation may similarly modulate oncogenic signaling pathways.

From a methodological perspective, our study demonstrates the utility of machine learning in developing a robust prognostic model based on palmitoylation-related features. Compared to traditional statistical methods, machine learning enables the efficient handling of large-scale, high-dimensional data and the identification of nonlinear, complex patterns [22]. By integrating multiomics data, we developed a comprehensive model that incorporates genomic, transcriptomic, and immune features, enhancing the accuracy of patient outcome predictions. Additionally, machine learning models can be adapted for clinical use, providing real-time, personalized treatment recommendations. Recent prognostic models in ccRCC report AUCs typically between 0.75 and 0.90 [23, 24]. In comparison, our model achieved AUCs of 0.96 (1 year), 0.98 (3 years), and 0.98 (5 years) in the TCGA cohort, demonstrating strong predictive performance despite using a relatively small set of genes.

In this study, functional enrichment analysis highlights the significant role of metabolic pathways, particularly lipid metabolism, in ccRCC prognosis. In addition to the identified pathways, palmitoylation's involvement in regulating lipid metabolism appears to be crucial in the pathogenesis of renal cancer. Studies have demonstrated that lipid metabolism is essential for the development and progression of renal cancer, with palmitoylation playing a pivotal role by modulating the function of lipid metabolism–related proteins [25]. For instance, palmitoylation may influence the proliferation and survival of ccRCC cells by regulating key enzymes in lipid metabolism pathways, thereby contributing to the metabolic reprogramming of tumor cells. This finding suggests that targeting palmitoylation-mediated lipid metabolism could offer novel therapeutic strategies for ccRCC, aligning with the observed metabolic alterations in the high-risk group. These results further underscore the critical intersection of palmitoylation, metabolic reprogramming, and tumor progression in ccRCC.

Our analysis also revealed that the high-risk group was associated with elevated TMB and CNV, both of which are established markers of aggressive cancer phenotypes and treatment resistance [26, 27]. Previous studies have demonstrated that a high TMB is associated with improved responses to immune checkpoint inhibitors, while certain CNVs are predictive of survival outcomes and disease progression [28, 29]. Therefore, a thorough understanding of both TMB and CNV is crucial for the development of personalized treatment strategies and the enhancement of prognostic models in clear ccRCC. Notably, our results also suggest that low-risk patients, identified through the palmitoylation-related prognostic model, are more likely to respond favorably to immune checkpoint inhibitors. This underscores the potential of palmitoylation as a valuable predictor of immunotherapy efficacy in ccRCC. Collectively, our findings highlight the complex interplay between metabolic reprogramming and immune modulation in ccRCC, emphasizing the importance of incorporating palmitoylation-related markers for improved prognosis prediction and treatment personalization.

Despite the promising results, several limitations should be acknowledged. First, the predictive performance of our model varied across the validation cohorts, which may be attributed to differences in sample size, patient heterogeneity, and cohort-specific clinical characteristics. Further validation in larger, independent, and prospectively collected cohorts is required to confirm the generalizability and clinical applicability of the model. Second, our study lacks direct functional validation of ZDHHC18-mediated palmitoylation targets and their downstream effects. Although our transcriptomic and bioinformatic findings are consistent with existing literature on the role of palmitoylation in cancer, additional in vitro and in vivo experiments are necessary to identify the specific substrates of ZDHHC18 in ccRCC and to elucidate their involvement in tumor progression, immune evasion, and therapy resistance. Finally, although the model demonstrates potential in predicting immunotherapy response, its clinical utility as a predictive biomarker for immunotherapy efficacy in ccRCC requires further validation in prospective clinical trials.

## Data Availability

The data that support the findings of this study are openly available in The Cancer Genome Atlas Program (TCGA) at https://portal.gdc.cancer.gov/, the International Cancer Genome Consortium (ICGC) at https://dcc.icgc.org/, the E-MTAB-1980 dataset at https://www.ebi.ac.uk/arrayexpress/, the Gene Expression Omnibus (GEO) under accession numbers GSE73731 and GSE40435 at https://www.ncbi.nlm.nih.gov/geo/, and the single-cell RNA sequencing data from the TISCH2 database at http://tisch.comp-genomics.org/.

## Supporting Information

Supporting Information 1 Figure S1: Independent prognostic significance of the palmitoylation-based risk score. (a) Univariate Cox regression analysis of the palmitoylation-related risk score and clinical variables, including age, T stage, N stage, M stage, grade, and overall stage. (b) Multivariate Cox regression analysis confirming that the risk score is an independent predictor of overall survival after adjustment for clinical covariates. (c) Calibration curve assessing the agreement between predicted and observed survival probabilities, demonstrating excellent model calibration. (d) Decision curve analysis (DCA) showing that the risk score provides superior net clinical benefit across a range of risk thresholds compared to conventional clinical parameters.

Supporting Information 2 Figure S2: Functional validation of ZDHHC18 knockdown in HK-2 cells. (a) Knockdown efficiency measured by qRT-PCR. (b) CCK-8 assay shows no significant proliferation change over 72 h. (c) Wound healing assay indicates comparable migration between the sh-ZDHHC18 and sh-NC groups.

Supporting Information 3 Table S1: List of palmitoylation-related genes.

Supporting Information 4 Table S2: Baseline clinical characteristics of ccRCC samples.

Supporting Information 5 Table S3: Experimental methods and protocols.
