# Supplementary figures and images for: Integrated Genomic and Functional Characterization of Palmitoylation in Clear Cell Renal Cell Carcinoma
Source: Hum Mutat. 2025 Nov 29;2025:4647115. doi: 10.1155/humu/4647115 (PMC12681404; doi:10.1155/humu/4647115)

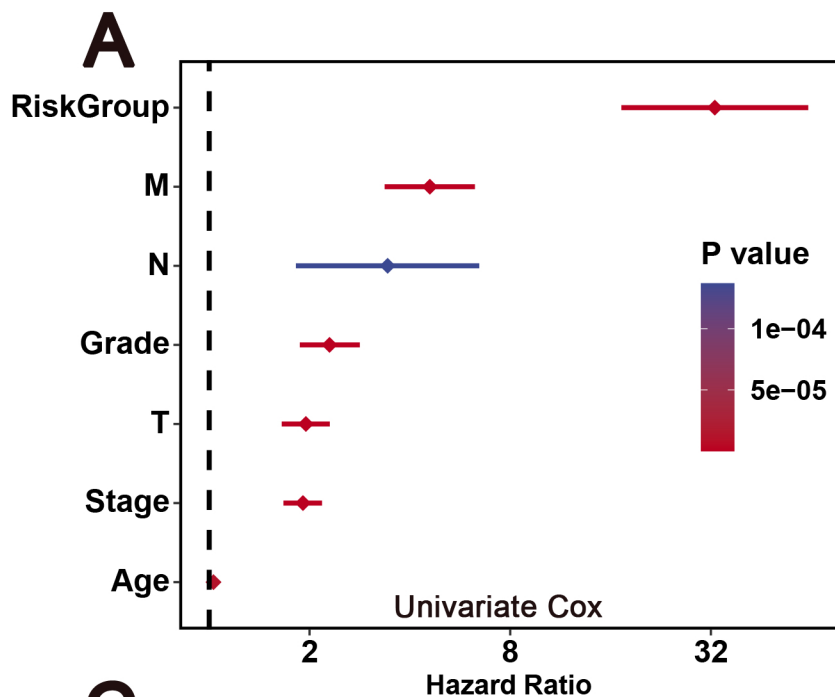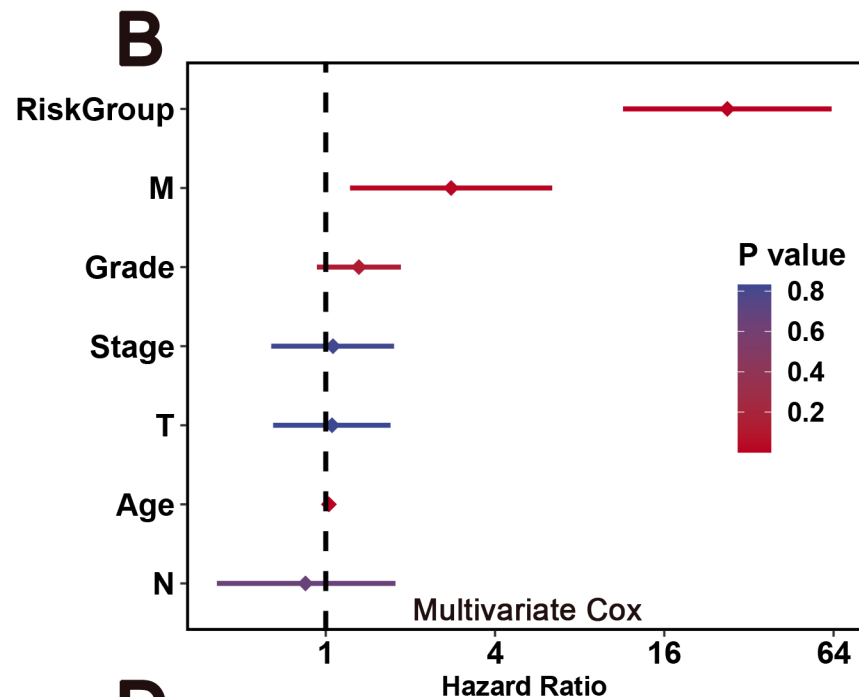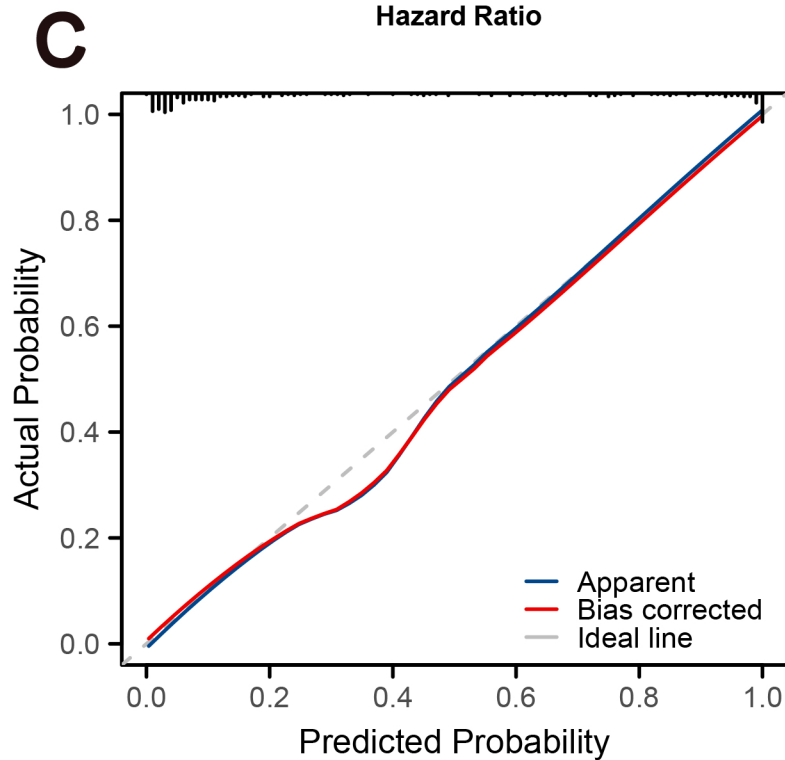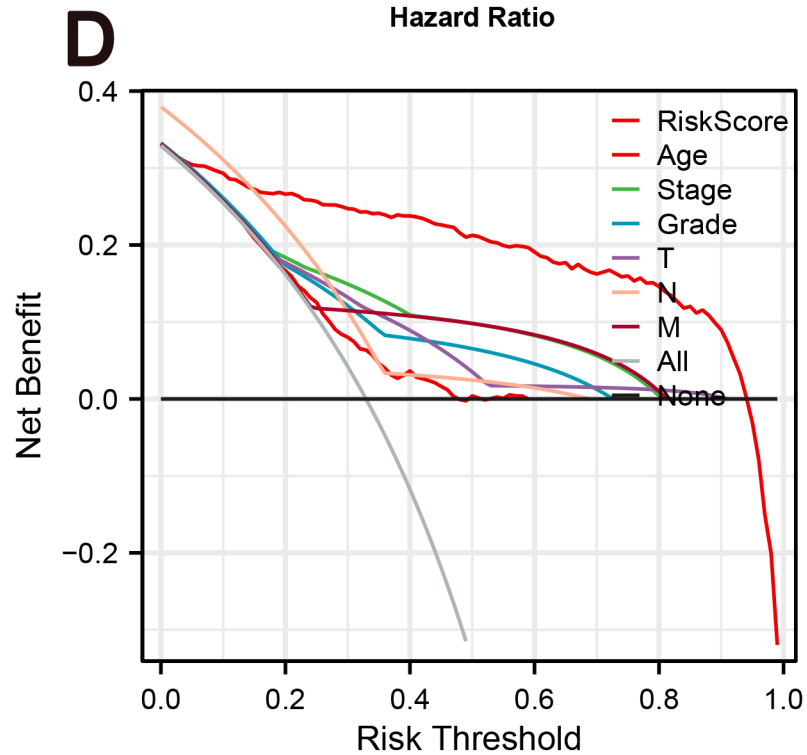

Supplement: Supporting Information 1 — Figure S1: Independent prognostic significance of the palmitoylation-based risk score. (a) Univariate Cox regression analysis of the palmitoylation-related risk score and clinical variables, including age, T stage, N stage, M stage, grade, and overall stage. (b) Multivariate Cox regression analysis confirming that the risk score is an independent predictor of overall survival after adjustment for clinical covariates. (c) Calibration curve assessing the agreement between predicted and observed survival probabilities, demonstrating excellent model calibration. (d) Decision curve analysis (DCA) showing that the risk score provides superior net clinical benefit across a range of risk thresholds compared to conventional clinical parameters. [file 4647115.f1.pdf]

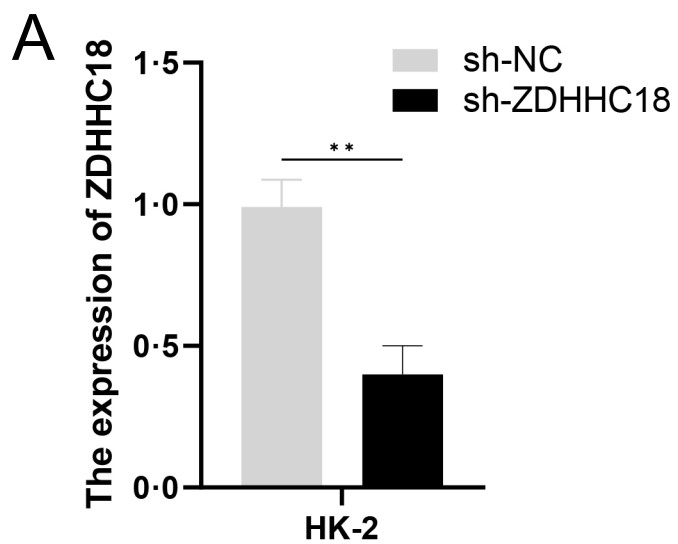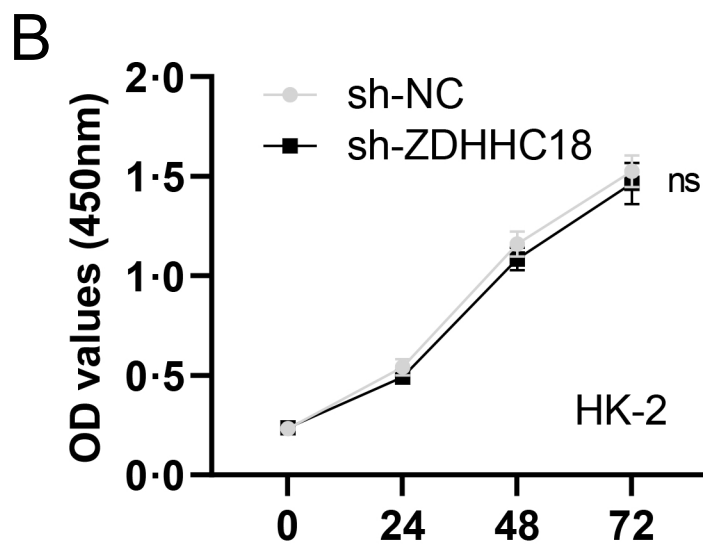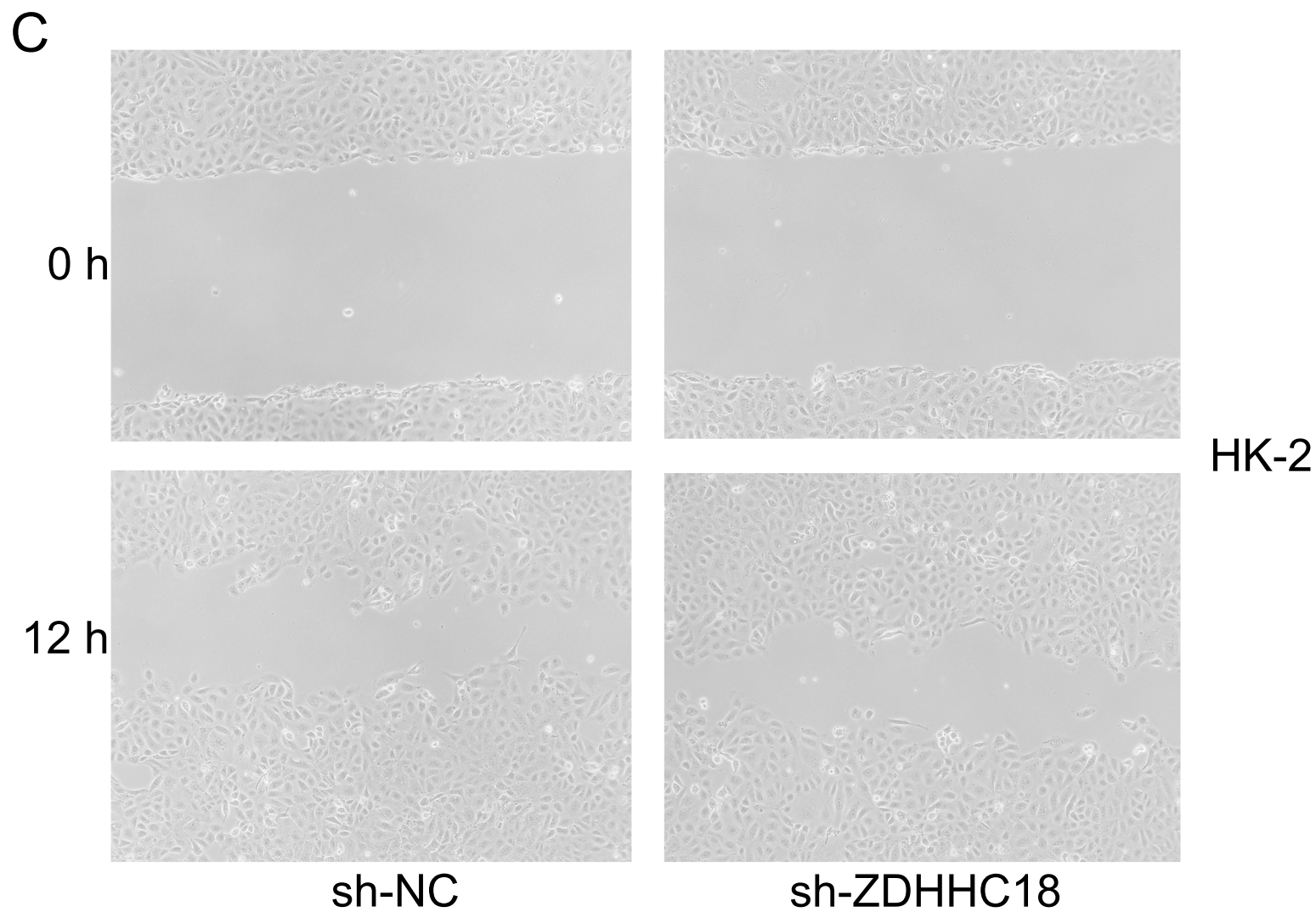

Supplement: Supporting Information 2 — Figure S2: Functional validation of ZDHHC18 knockdown in HK-2 cells. (a) Knockdown efficiency measured by qRT-PCR. (b) CCK-8 assay shows no significant proliferation change over 72 h. (c) Wound healing assay indicates comparable migration between the sh-ZDHHC18 and sh-NC groups. [file 4647115.f2.pdf]
