# Supplementary material for: Integrated Genomic and Functional Characterization of Palmitoylation in Clear Cell Renal Cell Carcinoma
Source: Hum Mutat. 2025 Nov 29;2025:4647115. doi: 10.1155/humu/4647115 (PMC12681404; doi:10.1155/humu/4647115)
Supplement: Supporting Information 4 — Table S2: Baseline clinical characteristics of ccRCC samples. [file 4647115.f4.docx]

**Supplementary Table 2. Baseline clinical characteristics of patients**

| Variables | Total (n = 1071) | E-MTAB-1980(n=101) | GSE40435 (n = 101) | GSE73731 (n = 265) | ICGC (n = 91) | TCGA (n = 513) | Statistic | *P* |
| --- | --- | --- | --- | --- | --- | --- | --- | --- |
|  |  |  |  |  |  |  |  |  |
|  |  |  |  |  |  |  |  |  |
| **Age, Mean ± SD** | 61.17 ± 11.54 | 63.48 ± 11.50 | 64.12 ± 9.24 | NaN ± NA | 60.47 ± 10.03 | 60.27 ± 12.06 | F=4.77 | **0.003** |
| **T stage, n(%)** |  |  |  |  |  |  | χ²=1088.91 | **<.001** |
| NA | 366 (34.17) | 0 (0.00) | 101 (100.00) | 265 (100.00) | 0 (0.00) | 0 (0.00) |  |  |
| T1 | 383 (35.76) | 68 (67.33) | 0 (0.00) | 0 (0.00) | 54 (59.34) | 261 (50.88) |  |  |
| T2 | 92 (8.59) | 11 (10.89) | 0 (0.00) | 0 (0.00) | 13 (14.29) | 68 (13.26) |  |  |
| T3 | 216 (20.17) | 21 (20.79) | 0 (0.00) | 0 (0.00) | 22 (24.18) | 173 (33.72) |  |  |
| T4 | 14 (1.31) | 1 (0.99) | 0 (0.00) | 0 (0.00) | 2 (2.20) | 11 (2.14) |  |  |
| **N stage, n(%)** |  |  |  |  |  |  | - | **<.001** |
| N0 | 402 (37.54) | 94 (93.07) | 0 (0.00) | 0 (0.00) | 79 (86.81) | 229 (44.64) |  |  |
| N1 | 21 (1.96) | 3 (2.97) | 0 (0.00) | 0 (0.00) | 2 (2.20) | 16 (3.12) |  |  |
| N2 | 4 (0.37) | 4 (3.96) | 0 (0.00) | 0 (0.00) | 0 (0.00) | 0 (0.00) |  |  |
| NA | 644 (60.13) | 0 (0.00) | 101 (100.00) | 265 (100.00) | 10 (10.99) | 268 (52.24) |  |  |
| **M stage, n(%)** |  |  |  |  |  |  | χ²=763.17 | **<.001** |
| M0 | 654 (61.06) | 89 (88.12) | 0 (0.00) | 53 (20.00) | 82 (90.11) | 430 (83.82) |  |  |
| M1 | 144 (13.45) | 12 (11.88) | 0 (0.00) | 44 (16.60) | 8 (8.79) | 80 (15.59) |  |  |
| NA | 273 (25.49) | 0 (0.00) | 101 (100.00) | 168 (63.40) | 1 (1.10) | 3 (0.58) |  |  |
| **Stage, n(%)** |  |  |  |  |  |  | χ²=716.92 | **<.001** |
| NA | 244 (22.78) | 0 (0.00) | 101 (100.00) | 140 (52.83) | 0 (0.00) | 3 (0.58) |  |  |
| Stage I | 410 (38.28) | 66 (65.35) | 0 (0.00) | 41 (15.47) | 48 (52.75) | 255 (49.71) |  |  |
| Stage II | 90 (8.40) | 10 (9.90) | 0 (0.00) | 12 (4.53) | 12 (13.19) | 56 (10.92) |  |  |
| Stage III | 180 (16.81) | 13 (12.87) | 0 (0.00) | 28 (10.57) | 22 (24.18) | 117 (22.81) |  |  |
| Stage IV | 147 (13.73) | 12 (11.88) | 0 (0.00) | 44 (16.60) | 9 (9.89) | 82 (15.98) |  |  |
| **Gender, n(%)** |  |  |  |  |  |  | - | **0.007** |
| Female | 383 (35.76) | 24 (23.76) | 42 (41.58) | 102 (38.49) | 39 (42.86) | 176 (34.31) |  |  |
| Male | 685 (63.96) | 77 (76.24) | 59 (58.42) | 160 (60.38) | 52 (57.14) | 337 (65.69) |  |  |
| NA | 3 (0.28) | 0 (0.00) | 0 (0.00) | 3 (1.13) | 0 (0.00) | 0 (0.00) |  |  |
| **Grade, n(%)** |  |  |  |  |  |  | χ²=965.61 | **<.001** |
| G1 | 69 (6.44) | 13 (12.87) | 22 (21.78) | 22 (8.30) | 0 (0.00) | 12 (2.34) |  |  |
| G2 | 415 (38.75) | 59 (58.42) | 47 (46.53) | 90 (33.96) | 0 (0.00) | 219 (42.69) |  |  |
| G3 | 342 (31.93) | 22 (21.78) | 24 (23.76) | 95 (35.85) | 0 (0.00) | 201 (39.18) |  |  |
| G4 | 135 (12.61) | 5 (4.95) | 8 (7.92) | 49 (18.49) | 0 (0.00) | 73 (14.23) |  |  |
| NA | 110 (10.27) | 2 (1.98) | 0 (0.00) | 9 (3.40) | 91 (100.00) | 8 (1.56) |  |  |
| F: ANOVA, χ²: Chi-square test, -: Fisher exact | | | | | | | | |
| SD: standard deviation | | | | | | | | |
