# Supplementary material for: Integrated Genomic and Functional Characterization of Palmitoylation in Clear Cell Renal Cell Carcinoma
Source: Hum Mutat. 2025 Nov 29;2025:4647115. doi: 10.1155/humu/4647115 (PMC12681404; doi:10.1155/humu/4647115)
Supplement: Supporting Information 5 — Table S3: Experimental methods and protocols. [file 4647115.f5.docx]

**Supplementary Table 3: Experimental Methods and Protocols**

**Cell culture and transfection**

Two ccRCC cell lines (786-O and Caki-1) and normal renal cells (HK-2) were obtained from the Cell Bank of the Chinese Academy of Sciences (Shanghai, China). 786-O and Caki-1 were maintained in RPMI-1640 medium (Gibco, USA). 10% fetal bovine serum (Gibco, USA), 1% penicillin and streptomycin (Gibco, USA) were added to the medium. HK-2 was maintained in DMEM/F-12 medium (BasalMedia, USA). 10% fetal bovine serum (Gibco, USA), 1% penicillin and streptomycin (Gibco, USA) were added to the medium. All were cultured at 37℃ in a humidified atmosphere containing 5% CO_2_.

To establish stable ZDHHC18 knockdown in cells, lentiviral vectors containing short hairpin RNA (shRNA) sequences targeting ZDHHC18 (shZDHHC18) were purchased from GenePharma (GenePharma, Shanghai, China). The knockdown efficiency was validated using qRT-PCR to ensure sufficient ZDHHC18 silencing. The shRNA sequences were: forward 5′-CCGGGTTTATTCTCTCCCTCTCATTCTCGAGAATGAGAGGGAGAGAATAAACTTTTTTG-3′ and reverse 5′-AATTCAAAAAAGTTTATTCTCTCCCTCTCATTCTCGAGAATGAGAGGGAGAGAATAAAC-3′.

**Quantitative real-time PCR (qRT-PCR)**

A previous study（PMID: 37985807）described specific methods for total RNA extraction and quantitative real-time PCR (qRT-PCR). The following primers were used in this study: ZDHHC18 forward, 5'-ACCGGCCTCTTCTTCGTCT-3' and ZDHHC18 reverse, 5'-AACTGCCTGTGTTGTCGATCT-3'; β-Actin forward, 5'-GAAGATCAAGATCATTGCTCCTC-3' and β-Actin reverse, 5'-ATCCACATCTGCTGGAAGG-3’. β-Actin was used as an internal control. The procedures were performed three times to ensure accuracy and precision. The relative expression levels were calculated using the 2^−ΔΔCT^ method.

**Cell Counting Kit-8 (CCK8) Assay**

Briefly, cells after different interventions were incubated in 96-well plates (2x10^3), supplemented with 100 µL culture medium and conditioned in 37℃ with 5% CO2. On days 1, 2 and 3, 10μL CCK-8 solution was added into each well, and incubation was performed for 2 h. Absorbance was measured at an optical density of 450 nm using a Microplate reader (Bio-Rad Laboratories, Inc.).

**Colony formation assay**

1000 cells were seeded into each well of a six-well plate and cultured in a humidified incubator with 5% CO2 for 10–14 days until visible colonies formed (>50 cells/colony). Cells were then washed with PBS (phosphate-buffered saline), fixed with 4% paraformaldehyde for 10 min, and stained with 0.5% crystal violet solution for an additional 20 min. Afterwards, the colonies were counted and examined. The assay was performed three times.

**5-Ethynyl-2’-deoxyuridine (EdU) assay**

The cells were evenly plated on a 96-well plate at a concentration of 10,000 cells/100 μl culture medium per well. After 24 hours, aspirate the culture medium in the wells, add 10 μM EdU reaction solution, and incubate at 37°C for 2 hours. Subsequently, it was fixed with 4% paraformaldehyde for 30 minutes, the membrane was broken with 0.5% Triton X-100, and 1×Apollo® reaction cocktail (100μL) was added for 30 minutes. The nuclei were then stained with 1×Hoechst 33342 for half an hour. Observe and take pictures under a fluorescence microscope.

**Transwell assay**

Transwell migration and invasion assays were performed in 24-well plate. After overnight serum starvation, 2x10^4^ 786-O or Caki-1 cells suspended in 200 µL serum-free medium were seeded into each insert, while the lower chamber was filled with 500 µL complete medium containing 10 % fetal bovine serum. For invasion experiments the upper surface of the insert was first coated with Matrigel and allowed to solidify at 37℃ for 30 min, whereas migration assays were conducted with uncoated inserts. Cells were incubated at 37℃ in a humidified 5 % CO_2_ atmosphere for 36 h (invasion) or 24 h (migration), after which inserts were rinsed with PBS, and non-migrated cells on the upper membrane surface were gently removed with a cotton swab. The membranes were then fixed in 4 % paraformaldehyde for 20 min, stained with 0.1 % crystal violet for 15 min.

**Wound Healing Assay**

Cells were seeded into 6-well plates and incubated until reaching full confluence. A sterile 200 μL yellow pipette tip was used to create a straight scratch across the cell monolayer. The wells were gently washed with PBS to remove detached cells. Subsequently, the cells were cultured in serum-free medium to eliminate the influence of proliferation. Images of the wound area were captured at 0 h and 12 h using an inverted microscope.
